# Replacing Fish Meal with Hydrolyzed Collagen Derived from Fish By-Products Improved Muscle Quality and Glycolipid Metabolism of Triploid Crucian Carp

**DOI:** 10.3390/foods12061235

**Published:** 2023-03-14

**Authors:** Fangle Tong, Jinhai Bai, Zhongtian Tang, Chunyan Li, Shaojun Liu, Zehong Wei

**Affiliations:** State Key Laboratory of Developmental Biology of Freshwater Fish, Engineering Research Center of Polyploid Fish Reproduction and Breeding of the State Education Ministry, College of Life Sciences, Hunan Normal University, Changsha 410081, China

**Keywords:** triploid crucian carp, fish meal, hydrolyzed collagen, muscle quality, glycolipid metabolism

## Abstract

Fish by-products are rich in collagen. Hydrolyzed collagen derived from fish by-products was used to replace fish meal to evaluate the effects on muscle quality and glycolipid metabolism of juvenile triploid crucian carp. A total of 240 juvenile fish with body weight of 10.01 ± 0.02 g were divided into four groups and fed four diets for 66 days: fish meal (FM) replaced with hydrolyzed collagen (HC) in 0% (Control), 2% (2% HC), 4% (4% HC), and 6% (6% HC), respectively. The results were as follows: The increased proportion of fish meal replaced with hydrolyzed collagen linearly and quadratically decreased the specific growth rate (SGR) of triploid crucian carp (*p* < 0.05). Compared with the control group, the SGR and intestinal α-amylase, trypsin and lipase activities in the 4% and 6% HC groups significantly decreased (*p* < 0.05), while there was no significant difference between the control and 2% HC groups (*p* > 0.05). Total umami amino acids content, chewiness and myofiber density of muscle in the 4% and 6% HC groups, as well as the essential fatty acids content in all HC groups increased significantly (*p* < 0.05). All HC groups significantly increased the serum glutathione peroxidase (GSH-Px) activity and decreased the serum malondialdehyde (MDA) content (*p* < 0.05). When the replacement amount reached 4%, the serum glucose and liver glycogen content, the liver and serum triglyceride (TG) content, and serum total cholesterol (T-CHO) content were significantly reduced (*p* < 0.05). In addition, the expression levels of insulin-like growth factor-1 (*IGF-1*) of the liver in all HC groups and lipolysis-related genes (lipoprotein lipase (*LPL*), carnitine O-palmitoyltransferase 1 (*CPT 1*) and hydroxyacyl-coenzyme A dehydrogenase (*HADH*)) of the liver in the 6% of HC group increased significantly (*p* < 0.05), and the expression levels of lipogenesis-related genes (fatty acid synthase (*FAS*) and sterol regulatory element-binding protein 1 (*SREBP 1*)) of the liver in the 4% HC and 6% HC groups decreased significantly (*p* < 0.05). In conclusion, the replacement of 2% fish meal with hydrolyzed collagen had no negative effects on the growth of triploid crucian carp, while the replacement of 4% fish meal with hydrolyzed collagen decreased SGR, but improved the muscle quality and decreased glycolipid levels. The maximum proportion of hydrolyzed collagen replacing fish meal should not exceed 4%.

## 1. Introduction

As a high-quality protein source for fish feed, fish meal is popular for its rich and balanced nutrients, easy digestibility and palatability [1]. With the rapid development of global fisheries, aquaculture has seen an increasing demand for fish meal, which is mainly composed of dried fish carcasses [1,2]. Dependence on fish meal exerts enormous pressure on limited marine resources and finding alternative resources of fish meal remains urgent [2]. The alternative resources of fish meal can be obtained not only from animals but also from plants. However, compared with fish meal, plant protein sources have some defects, such as anti-nutritional factors enrichment and amino acid imbalance [3,4]. Animal protein sources are the ideal component in animal feed formulation, and they are superior to plant protein sources [1]. Some animal protein sources, such as meat and bone meal (MBM), hydrolyzed animal protein and blood meal (BM), are commonly used. It was reported that no significant difference was discovered in the specific growth rate (SGR) of large yellow croaker (*Pseudosciaena crocea*) fed with MBM replacing 45% fish meal [5]. In juvenile Nile tilapia (*Oreochromis niloticus*), up to 50% of fish meal could be replaced with hemoglobin powder, which did not affect SGR [6]. Dietary 12.5% hydrolyzed feather meal (76% fish meal replacement) was possible in European seabass (*Dicentrarchus labrax*) without growth decline [7]. Notably, there were many studies on fish meal replaced with various animal protein sources in aquatic feed, while few studies were carried out on hydrolyzed collagen. 

At present, three main sources are used to extract hydrolyzed collagen: terrestrial animal by-products, leather waste and fish by-products [1,8,9]. However, infectious diseases existing in terrestrial animal by-products (such as foot-and-mouth disease) and chromium in leather waste limit the extraction of hydrolyzed collagen from the above two raw materials [1,8], thus fish by-products are gradually welcomed by the public. Collagen is abundant in fish by-products (such as fish skin and bone) [10]. In deep-sea redfish (*Sebastes mentella*), the collagen content in skin, scale and bone were 47.5%, 6.8% and 10.3%, respectively [9]. In carp (*Cyprinus carpio*), the yields of acid-soluble collagens in skin, scale and bone were 41.3%, 1.35% and 1.06%, respectively [11]. Fish by-products account for about 25% of the global harvest, of which 13% is used for fish meal production, and the rest is discarded [12]. The discarded fish by-products not only pollute the environment but also waste resources. The key to extracting hydrolyzed collagen from fish by-products is to break the peptide bonds of the collagen, which can be achieved using heat, acids, bases, enzymes or a combination of these physical, chemical and biological methods [13]. Hydrolyzed collagen obtained from fish by-products consists of low molecular weight peptides such as Pro-Hyp [14]. The Pro-Hyp is proven to improve the dysfunction of the skin barrier and promote the growth and differentiation of skin fibroblasts [15]. In addition, hydrolyzed collagen helps to lower blood pressure, improve immunity and promote calcium absorption [10]. However, the investigation of the effect of hydrolyzed collagen on fish muscle quality and glycolipid metabolism remains insufficient.

Triploid crucian carps (3n = 150) were obtained from crossing the male allotetraploid (4n = 200) (intercrossing between *Carassius auratus* red var. (♀) and *Cyprinus carpio* L. (♂)) with the female Japanese crucian carp (*Carassius auratus cuvieri*) (2n = 100) [16]. The triploid crucian carp is sterile, and its sterility makes it possible for reproductive energy to be transferred to growth performance [17]. Therefore, triploid crucian carps are expected to show the advantages of faster growth and longer life span [17]. The sterility of triploid crucian carp also hinders them from mating with other fish in nature, which is of great significance for safeguarding fish genetic resources [16]. In addition, the advantages of strong disease resistance and good quality flesh of triploid crucian carp make it readily accepted by customers and farmers [16]. As far as we know, few studies have been carried out on the feeding and nutrition of triploid crucian carp. In the present study, we aimed to explore the effects of fish meal replaced with hydrolyzed collagen on the growth performance, muscle quality and glycolipid metabolism of triploid crucian carp. This will contribute to the application of fish by-products and the development of an environmentally friendly economy. 

## 2. Materials and Methods

### 2.1. Experimental Diets

The Animal Ethics Experimental Committee of Hunan Normal University (Changsha, China) ratified all the conduct of this study. The main protein sources of the diets were fish meal, soybean meal, rapeseed meal and wheat flour. The main lipid source of the diets was soybean oil. Four isonitrogenous (32% crude protein) and isolipidic (8% crude lipid) diets were designed to replace fish meal with 0% (Control), 2% (2% HC), 4% (4% HC), and 6% (6% HC) hydrolyzed collagen, respectively. The experimental diets of triploid crucian carp are displayed in Table 1. Hydrolyzed collagen (purity: 99%) extracted from flesh and by-products of tilapia (*Oreochromis niloticus*) was from Baichuan Biotechnology Co., Ltd. (Xi’an, China). The ingredients were mixed evenly according to the principle of step-by-step magnification and finally made into floating pellets with a diameter of 1 mm. The dried pellets were stored at −20 °C until use.

The comparison of proximate composition between hydrolyzed collagen and fish meal is displayed in Table 2. The crude protein content of hydrolyzed collagen was 88.51%, which was about 1.6 times that of fish meal. The crude lipid content of fish meal was 10.6%, which was about 1.53 times that of hydrolyzed collagen.

The comparison of amino acid content between hydrolyzed collagen and fish meal is displayed in Table 3. The total amino acid content of hydrolyzed collagen was 93.26%, which was higher than that of fish meal (56.10%). However, the content of total essential amino acids of hydrolyzed collagen was 23.18%, which was lower than that of fish meal (26.21%). It can be calculated from the table that the methionine and isoleucine content of fish meal were about 1.65 and 1.99 times higher than those of hydrolyzed collagen, respectively. Both glycine and proline content in hydrolyzed collagen were about 5 times higher than those of fish meal, while hydroxyproline content was about 18.59 times as much as fish meal.

The amino acid composition of diets is displayed in Table 4. The total essential amino acid content in the 4% HC and 6% HC groups were 12.7% and 11.99%, respectively, which were lower than those in the control group (13.25%) and 2% HC group (13.61%). The content of eight kinds of essential amino acids (methionine, valine, lysine, isoleucine, phenylalanine, leucine, threonine and histidine) in the 4% and 6% HC groups were lower than those in the control group and the 2% HC group.

### 2.2. Feeding Trial

Triploid crucian carp were obtained at the Engineering Center of Polyploidy Fish Breeding of the National Education Ministry, Hunan Normal University, Hunan, China. The experiment was implemented in the circulating aquaculture system of Hunan Agricultural University (Changsha, China). Before the experiment, the juvenile triploid crucian carp were domesticated with the control feed for two weeks. A total of 12 tanks with 20 fish (10.01 ± 0.02 g fish^−1^) in each tank (volume 400 L tank^−1^) were set up. The 12 tanks were divided into 4 treatment groups, and every 3 tanks belong to the same treatment group. The water in the tank was changed once a week. Aquariums were equipped with oxygenation pumps and the lamp was used as the source of light (12 h light: 12 h dark). Fish were fed to apparent satiation at 6:00, 12:00 and 17:30, respectively. The feeding trial was conducted for 66 days. The water conditions of the feeding trial were shown as follows: water temperature was 25.3 ± 3.5 °C, pH was 7.4 ± 0.4 and dissolved oxygen was 7.0 ± 0.5 mg/L.

### 2.3. Sample Collection

At the end of the feeding trial, all fish were fasted for 24 h and anesthetized with ethyl 3-aminobenzoate methyl sulfonate (MS-222, Sigma, St. Louis, MO, USA) at a concentration of 100 ppm. Fish in each aquarium were counted and weighed to evaluate the growth performance. The length and weight of the body and the weight of the liver and viscera of fish were recorded for analysis of morphometric indexes (three fish per aquarium). Blood was obtained from the tail vein of fish with syringes, and stored in a refrigerator at 4 °C overnight. Subsequently, the blood was centrifuged (4 °C, 3500 r/min, 15 min) to obtain serum for biochemical analysis. Fish were dissected on ice. One side of the dorsal muscle (1 cm × 0.5 cm × 0.5 cm) in the same position of each fish was immersed in 4% paraformaldehyde for histomorphological analysis, and the other side of the dorsal muscle in the same position of each fish was used for texture and pH measurement (three fish per replicate). The rest of the muscle, liver and gut used for biochemical analysis were put into liquid nitrogen irrigation as soon as possible and stored at −80 °C. 

### 2.4. Growth Performance and Morphometric Indexes Analysis 

Weight gain rate (WGR), specific growth rate (SGR), condition factor (CF), hepatosomatic index (HSI), and viscerosomatic index (VSI) were calculated according to the method of the previous studies [5,6].

WGR (%) = (W_af_ − W_ai_)/W_ai_

SGR (%/d) = 100 × [Ln (W_af_) − Ln (W_ai_)]/d

CF (g/cm^3^) = 100 × W_f_/L_b_^3^

HSI (%) = 100 × W_l_/W_f_

VSI (%) = 100 × W_v_/W_f_

where W_af_ represents average final body weight, W_ai_ represents average initial body weight, d represents feeding days, W_f_ represents final body weight of a fish, L_b_ represents body length of a fish, W_l_ represents liver wet weight of a fish, and W_v_ represents viscera wet weight of a fish.

### 2.5. The Diet and Muscle Composition Analysis

#### 2.5.1. Proximate Compositions Analysis

The muscle and diets were dried to constant weight using the freeze dryer to determine moisture. Crude protein and crude lipid content of muscle and diets were measured according to the method of the Association of Analytical Communities (AOAC, 2005). Kjeltec auto analyzer (Kjeltec 2300, FOSS, Stockholm, Sweden) and soxhlet extractor (Soxtec 2050, FOSS, Sweden) were used to determine crude protein and crude lipid content, respectively.

#### 2.5.2. Hydrolyzed Amino Acid

The content of hydrolyzed amino acids in the raw materials and diets was measured according to the method of the previous study [18]. About 0.05 g diet (dry matter) (about 0.025 g fish meal/hydrolyzed collagen) weighed and recorded was put into hydrolysis tubes with 5 mL HCl (6 M) added. Then, the hydrolysis tube filled with nitrogen (N_2_) was digested at 110 °C for 24 h. After digestion, the solution in the hydrolysis tube was transferred to a 50 mL volumetric flask and ultra-pure water was replenished. Subsequently, 1 mL of solution was removed from the volumetric flask and dried in a nitrogen-filled condition. After the dry matter was dissolved with 1 mL HCl (0.02 M), a 0.22 μm ultrafiltration membrane (Millipore, Billerica, MA, USA) was utilized to filter the solution, and the high-speed amino acid analyzer (L-8900, Hitachi, Tokyo, Japan) was utilized to determine the hydrolyzed amino acids of the solution.

#### 2.5.3. Free Amino Acid

The content of free amino acids in muscle was determined according to the method of the previous study [19]. Approximately 0.5 g of muscle (wet weight) weighed and recorded was put into a centrifuge tube with 3 mL sulfosalicylic acid (10%) added. After homogenization and centrifugation (13,000 rpm, 4 °C, 15 min), a 0.22 µm ultrafiltration membrane (Millipore, MA, USA) was utilized to filter the supernatant, and the high-speed amino acid analyzer (LA8080, HITACHI, Japan) was utilized to determine the free amino acid.

#### 2.5.4. Fatty Acid Composition

About 1 g of muscle (dry matter) was added to 10 mL of Folch solution for homogenization. The homogenate filled with nitrogen was shaken by ultrasound for 20 min and centrifuged (2500 r/min) for 10 min. Subsequently, the liquid in the underlayer was dried at 50 °C, filled with N_2_ and then added with 2 mL of potassium hydroxide (KOH) solution. The solution filled with N_2_ was incubated in water at 50 °C until the oil droplets disappeared and then added with 2 mL of boron trifluoride (BF_3_) solution. The mixture solution filled with N_2_ was incubated in water at 50 °C for 3 min and then added with normal heptane. Saturated saline was added to wash the upper layer. Subsequently, the supernatant was added with a little anhydrous sodium sulfate for gas chromatography analysis. The fatty acids composition of muscle was measured using the Varian column (FAME CP-sil88, 0.25 mm × 50 m × 0.20 μm). The identification of fatty acids was based on the gas chromatogram of fatty acid standard, and the method of peak area normalization was used to calculate the relative percentage content of fatty acids. 

### 2.6. Muscle pH, Texture and Histology Analysis

The pH meter (Testo-205, Testo AG, Lenzkirch, Germany) was used to analyze the muscle pH value according to the method of the previous study [20]. Three tests were performed in each sample.

A texture analyzer (Food Technology Corporation, Sterling, VA, USA), which was equipped with an 8 mm cylinder probe and a 250 N weighing cell, was utilized to measure muscle texture according to the method of the previous study [21]. A double compression experiment with a compression ratio of 60% was carried out. During the experiment, the moving speed of the probe was 1 mm/s, and 2 s after the end of the first compression, the second compression was implemented. Hardness, adhesiveness, cohesiveness, springiness and chewiness of muscle were determined using a texture analyzer.

For histological analysis [22], muscle samples soaked in paraformaldehyde were dehydrated step by step in ethanol and xylene. Subsequently, paraffin wax was utilized to embed the dewatered samples. After the samples were sliced, hematoxylin and eosin (HE) were utilized to stain. The optical microscope, which was equipped with a camera system (BX40F4, Olympus, Tokyo, Japan) was utilized to observe the morphology of muscle and photograph. The diameter and density of myofiber were measured and calculated by ImageJ software according to the previous methods [22].

### 2.7. Serum, Intestinal and Liver Enzymes Activities Measurement

The assay kits, which were purchased from Nanjing Jiancheng Bioengineering Institute (Nanjing, China), were utilized to determine the activities of catalase activity (CAT), superoxide dismutase activity (SOD), glutathione peroxidase (GSH-Px), α-amylase, trypsin, and lipase, and the content of malondialdehyde (MDA), glucose, glycogen, triglyceride (TG), total cholesterol (T-CHO), high-density lipoprotein cholesterol (HDL-C) and low-density lipoprotein cholesterol (LDL-C) following the manufacturer’s specification strictly.

### 2.8. Real-Time Quantitative PCR Analysis

The RNA isolator total RNA Extraction Reagent (Vazyme, R401-01) was utilized to extract total RNA. After the determination of quality, concentration and integrity of the total RNA, the reagent of All-in-one RT SuperMix Perfect for qPCR (Vazyme, R33) was utilized to reversely transcribe total RNA into cDNA. Real-time quantitative PCR analysis was implemented with the ChamQ Universal SYBR qPCR Master Mix (Vazyme, Q711-03). Primer Premier 5 was used for primers design, and the specific primer sequences are exhibited in Table 5. The total volume of the reaction was 20 µL and the amplification conditions were as follows: 40 cycles of 95 °C for 15 s and 60 °C for 1 min. At the end of the PCR reaction, dissolution curve analysis was performed, and the data were converted into Ct values. The expression of the target gene relative to *β-actin* was calculated according to the equation of R = 2^−ΔΔCt^ [23].

### 2.9. Statistical Analysis

The software of SPSS 17.0 was utilized to analyze data. All the data were expressed as the mean ± standard error (SE). The method of one-way ANOVA and the test of Tukey were utilized to determine the significance of different research groups. In addition, orthogonal polynomial contrasts were performed to determine whether the effect was linear and/or quadratic. The significant difference limen was set at 0.05.

## 3. Results

### 3.1. Growth Performance and Morphometric Indexes

The growth performance and morphometric indexes of triploid crucian carp are illustrated in Table 6. The increased proportion of fish meal replaced with hydrolyzed collagen linearly and quadratically decreased the SGR and WGR of triploid crucian carp (*p* < 0.05). Compared with the control group, the 2% HC group had not significantly changed SGR and WGR (*p* > 0.05), while the 4% HC and 6% HC groups had significantly lower WGR and SGR (*p* < 0.05). The influence of replacing fish meal with hydrolyzed collagen on HSI, VSI and CF of triploid crucian carp was not significant (*p* > 0.05).

### 3.2. Muscle Nutritional Composition

The muscle nutrition components are shown in Table 7. No significant differences were observed in muscle moisture, crude protein and crude lipid content among all the treatments (*p* > 0.05).

Muscle free amino acid content of fish is listed in Table 8. Compared with the control group, the 4% HC and 6% HC groups had significantly higher content of total umami amino acids (*p* < 0.05), and all HC groups had significantly lower content of total bitter amino acids (*p* < 0.05). The total sweet amino acids content of muscle in the 4% HC group was significantly lower than that in other groups (*p* < 0.05).

The muscle fatty acid composition of fish is listed in Table 9. The total saturated fatty acids (SFAs) content of muscle in all HC groups decreased significantly compared with that in the control group (*p* < 0.05). No significant difference was detected in total monounsaturated fatty acids (MUFAs) content among all the treatments (*p* > 0.05). Compare with the control group, the 4% HC group had significantly higher content of total polyunsaturated fatty acids (PUFAs) (*p* < 0.05), and all HC groups had significantly higher content of total n-6 polyunsaturated fatty acids (n-6 PUFAs) (*p* < 0.05). The content of α-linolenic acid (ALA, C18:3 n-3) in 2% HC and 4% HC groups and the linoleic acid (LA, C18:2 n-6) in all HC groups increased significantly compared with those in the control group (*p* < 0.05). 

### 3.3. Muscle Texture, pH Value and Histology

Muscle texture and pH value are displayed in Table 10. The proportion of fish meal replaced with hydrolyzed collagen linearly and quadratically affected the muscle hardness, chewiness and adhesiveness of triploid crucian carp (*p* < 0.05).Muscle hardness in the 6% HC group and muscle chewiness in the 4% and 6% HC groups were significantly higher than those in the control group (*p* < 0.05). Compared with the control group, muscle adhesiveness in all HC groups decreased significantly (*p* < 0.05). There were no significant effects in muscle cohesiveness, springiness and pH value of fish with different treatments (*p* > 0.05).

**Figure 1:**
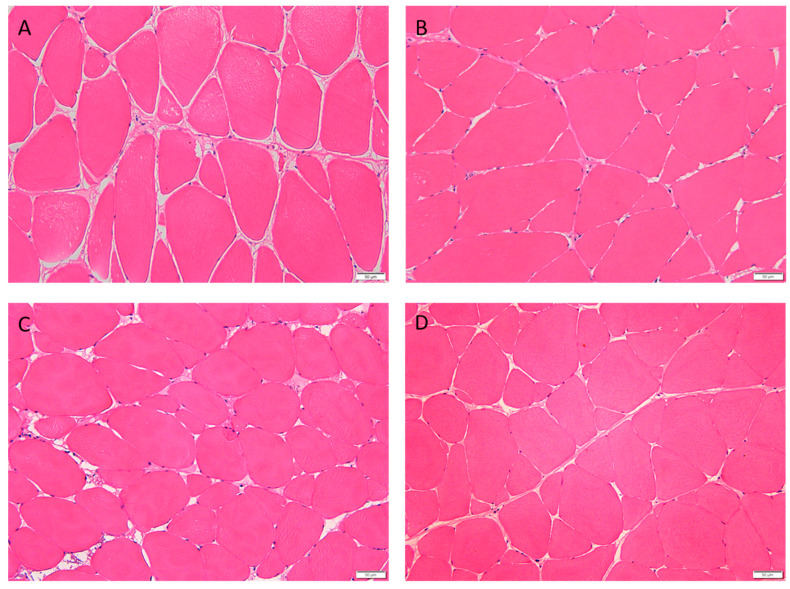
Muscle histology of triploid crucian carp fed with different diets (200×). (**A**): Control group; (**B**): 2% HC group; (**C**): 4% HC group; (**D**): 6% HC group.

Muscle morphology is shown in Figure 1, and myofiber density and diameter are listed in Table 10. The increased proportion of fish meal replaced with hydrolyzed collagen linearly and quadratically increased myofiber density of triploid crucian carp (*p* < 0.05).The myofiber densities in the 4% HC and 6% HC groups were significantly higher and the myofiber diameters were significantly lower than those in the 2% HC group and the control group (*p* < 0.05). 

### 3.4. Serum, Intestinal and Liver Enzymes Activities

Effects of hydrolyzed collagen replacing fish meal on serum, intestinal and liver enzyme activities of triploid crucian carp are listed in Table 11. The influence of replacing fish meal with hydrolyzed collagen on serum CAT activity was not significant (*p* > 0.05). The effects of hydrolyzed collagen replacing fish meal on GSH-Px activity and MDA content in the serum of triploid crucian carp were linear and quadratic, and quadratic on SOD activity (*p* < 0.05). Compared with the control group, the 6% HC group had a significantly higher activity of serum SOD (*p* < 0.05), and all HC groups had a significantly higher activity of serum GSH-Px (*p* < 0.05) and significantly lower content of serum MDA (*p* < 0.05). The increased proportion of fish meal replaced with hydrolyzed collagen linearly and quadratically decreased the activities of intestinal α-amylase and lipase of triploid crucian carp (*p* < 0.05). The activities of intestinal α-amylase, trypsin and lipase decreased significantly when the proportion of fish meal replaced with hydrolyzed collagen reached 4% (*p* < 0.05). The effects of hydrolyzed collagen replacing fish meal on serum glucose and hepatic glycogen content, serum TG, T-CHO and LDL-C content, and liver TG content of triploid crucian carp were linear and quadratic (*p* < 0.05). Compared with the control group, the 4% and 6% HC groups had significantly lower content of serum glucose and hepatic glycogen (*p* < 0.05), and serum TG and T-CHO content in all HC groups and serum LDL-C content in 6% HC group decreased significantly (*p* < 0.05). The liver TG content decreased significantly with the proportion of fish meal replaced with hydrolyzed collagen up to 4% (*p* < 0.05). There were no significant differences in liver T-CHO and serum HDL-C content among all the treatments (*p* > 0.05).

### 3.5. Expression Levels of Genes Related to Glucose Metabolism 

**Figure 2:**
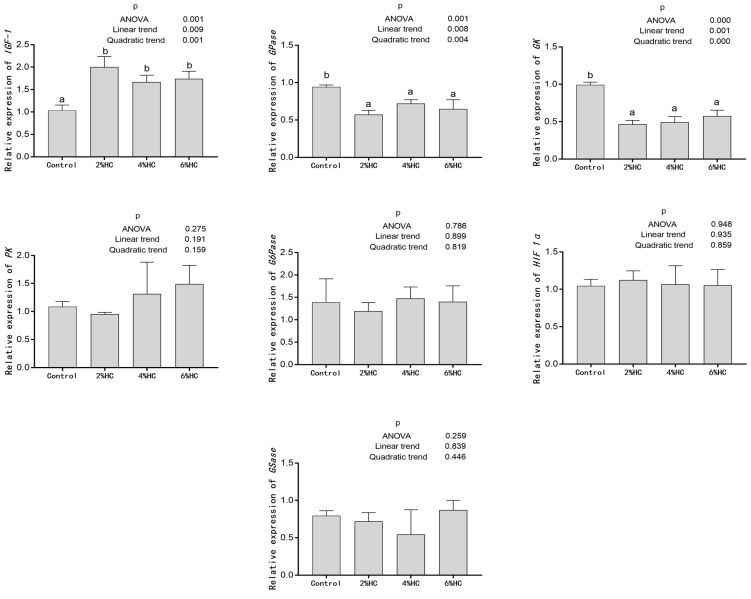
Effects of hydrolyzed collagen replacing fish meal on the expression levels of genes related to glucose metabolism in liver of triploid crucian carp. Values are presented as mean ± SE (*n* = 3). Different letters indicate significant differences (*p* < 0.05).

Effects of hydrolyzed collagen replacing fish meal on the expression levels of genes related to glucose metabolism in the liver of triploid crucian carp are displayed in Figure 2. Positive linear and quadratic trends were observed between the proportion of hydrolyzed collagen replacing fish meal and expression levels of insulin-like growth factor-1 (*IGF-1*), glucokinase (*GK*) or glycogen phosphorylase (*GPase*) in the liver (*p* < 0.05). Compared with the control group, the relative expression level of *IGF-1* gene in all HC groups increased significantly (*p* < 0.05), and the relative expression levels of *GK* and *GPase* genes in all HC groups decreased significantly (*p* < 0.05). There were no significant differences in the relative expression levels of pyruvate kinase (*PK*), glucose-6-phosphatase (*G6Pase*), hypoxia-inducible factor 1α (*HIF 1α*), and glycogen [starch] synthase (*GSase*) genes among all the treatments (*p* > 0.05).

### 3.6. Expression Levels of Genes Related to Lipid Metabolism

**Figure 3:**
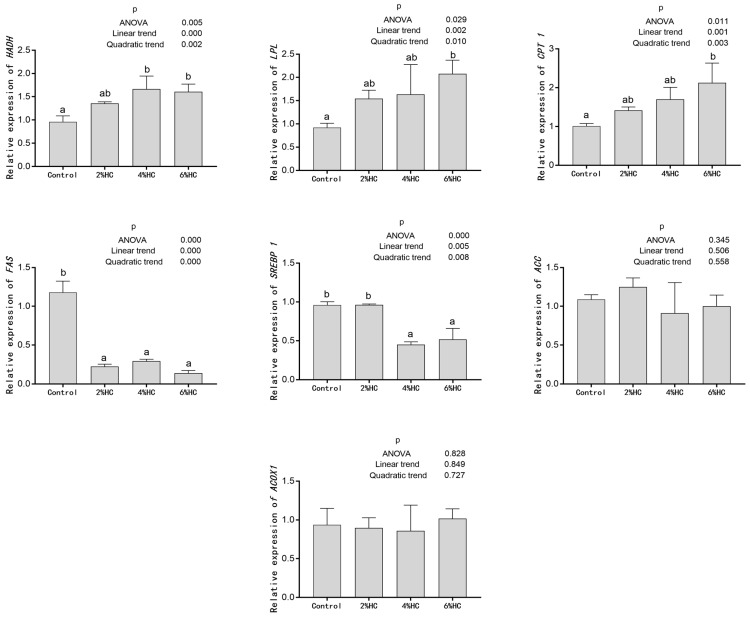
Effects of hydrolyzed collagen replacing fish meal on the expression levels of genes related to lipid metabolism in liver of triploid crucian carp. Values are presented as mean ± SE (*n* = 3). Different letters indicate significant differences (*p* < 0.05).

Effects of hydrolyzed collagen replacing fish meal on the expression levels of genes related to lipid metabolism in the liver of triploid crucian carp are shown in Figure 3. The increased proportion of fish meal replaced with hydrolyzed collagen linearly and quadratically increased the expression levels of hydroxyacyl-coenzyme A dehydrogenase (*HADH*), lipoprotein lipase (*LPL*) and carnitine O-palmitoyltransferase 1 (*CPT 1*) in the liver of triploid crucian carp (*p* < 0.05).The relative expression levels of *LPL* and *CPT 1* genes in the 6% HC group, and *HADH* gene in 4% HC and 6% HC groups increased significantly compared with those in the control group (*p* < 0.05). The increased proportion of fish meal replaced with hydrolyzed collagen linearly and quadratically decreased the expression levels of fatty acid synthase (*FAS*) and sterol regulatory element-binding protein 1 (*SREBP 1*) in the liver of triploid crucian carp (*p* < 0.05).The relative expression levels of the *FAS* gene in all HC groups, and *SREBP 1* gene in 4% HC and 6% HC groups decreased significantly compared with those in the control group (*p* < 0.05). The impacts of replacing fish meal with hydrolyzed collagen on the expression levels of acyl-CoA oxidase 1 (*ACOX1*) and acetyl-CoA carboxylase (*ACC*) genes were not significant (*p* > 0.05).

## 4. Discussion

Fish by-products, containing some bioactive peptides (such as Hyp-Pro), are one of the main sources of collagen extraction [10,11]. In the present study, the crude protein content of hydrolyzed collagen extracted from by-products of tilapia was as high as 88.51%, while that of fish meal was 55.25%. The content of glycine, alanine, arginine and proline in hydrolyzed collagen was higher than those in fish meal. Glycine and alanine have the effect of stimulating fish feeding [24]. Arginine, as a precursor, is involved in the synthesis of urea, polyamines, agmatine, proline and glutamate [25]. Proline is proven to be crucial in collagen synthesis and cell differentiation [26]. In the present research, the WGR and SGR of fish decreased significantly when the proportion of fish meal replaced with hydrolyzed collagen reached 4%. There might be two reasons for the decline of SGR and WGR. One was the lower content of essential amino acids of hydrolyzed collagen. In the present study, the EAAs content in fish meal and hydrolyzed collagen were 26.21% and 23.18%, respectively. With the increase in the proportion of hydrolyzed collagen replacing fish meal, the content of essential amino acids in 4% and 6% HC groups decreased. The content of methionine, valine, lysine, isoleucine, phenylalanine, leucine, threonine and histidine in the 4% and 6% HC groups were lower than those in the control group and 2% HC group, which may cause the nutritional requirements of triploid crucian carp in 4% and 6% HC groups to not be satisfied. Ai et al. [5] found that the decline of the growth of large yellow croaker was related to the imbalance of essential amino acids in MBM. The other was the decreased digestion ability of fish to feed. The activities of intestinal amylase, trypsin and lipase decreased significantly when the proportion of fish meal replaced with hydrolyzed collagen reached 4%. Starch, protein and fat in food are hydrolyzed under the action of intestinal amylase, trypsin and lipase, respectively [27], which help nutrients in food to be absorbed by the body. In the present experiment, the SGR of triploid crucian carp decreased significantly when the proportion of fish meal replaced with hydrolyzed collagen reached 4%, and there were no significant differences in morphometric indexes and nutrition components of muscle among all the treatments. The above results were in conformity with some former research. Zhao et al. [28] found that when 4% hydrolyzed collagen was added to the feed, it significantly decreased the SGR of grass carp (*Ctenopharyngodon idellus*), while affecting muscle nutrition components in a not significant way. There was no significant difference in liver weight between mice fed with a normal diet and oral administration of fish collagen hydrolysates for 14 days, respectively [8].

Umami, sweet, and bitter tastes are closely related to consumers’ acceptance or rejection of food [29]. Glutamates releases a unique taste, which is the original definition of umami taste [30]. The α-glutamyl dipeptides and tripeptides, especially peptides containing aspartic acid, threonine and serine, present a taste of umami, and the umami molecules endow salty taste and increase the intensities of other tastes [29]. The taste of sweetness is largely related to the content of glycine and alanine [30]. The name “glycine” comes from the Greek word “glykys”, which means sweet, and its sweetness is similar to glucose [26]. Bitter food is usually rejected by consumers, however, limited bitterness in food may be desirable [29]. The increased glutamic acid content in 4% HC and 6% HC groups and serine content in all HC groups might be related to the fact that hydrolyzed collagen was rich in proline and glycine, which could be converted into glutamic acid and serine, respectively [26,31]. The free proline content of muscle in all HC groups decreased significantly, which might be related to the conversion of proline into glutamic acid and other amino acids [31]. The arginine content in hydrolyzed collagen was higher than that in fish meal, while the free arginine content of muscle in all HC groups decreased significantly with hydrolyzed collagen replacement, which needed to be further studied. The results that the replacement of fish meal with hydrolyzed collagen significantly increased the umami amino acids content and significantly decreased the bitter amino acids content of muscle showed that hydrolyzed collagen was helpful to improve the taste of the triploid fish. 

Fatty acids are hydrocarbon chains with 2–36 carbon atoms, one end of which is methyl and the other end is carboxyl [32]. As the substantial components of lipids, fatty acids are important energy substrates, meanwhile, they are the components of phospholipids involved in the formation of cell membranes [33]. Fatty acids can be divided into two categories: saturated fatty acids (SFAs) without double bonds and unsaturated fatty acids with double bonds. Unsaturated fatty acids families include n-3, n-6 and n-9 families, and the position of the first double bond is different among these three fatty acids [32]. Human can synthesize most kinds of fatty acids, except for α-linolenic acid (ALA, C18:3 n-3) and linoleic acid (LA, C18:2 n-6), because human lacks the desaturase enzymes that catalyze the formation of the double bond at the n-3 or n-6 position of the hydrocarbon chain (calculating from the methyl carbon), respectively [34]. The ALA and LA can only be obtained from food, therefore, they are termed essential fatty acids [35]. When the uptake of SFAs increases, the LDL-C content in the body increases, which makes the human body more prone to coronary heart disease [36]. The n-3 PUFAs are vital in decreasing lipogenesis, and the n-6 PUFAs have certain benefits in decreasing total cholesterol, LDL-C and HDL-C content [33]. In the present research, the ALA and LA content increased significantly and the SFAs content decreased significantly with hydrolyzed collagen replacement, indicating that hydrolyzed collagen was beneficial in improving fatty acids composition of fish muscle.

Texture profile analysis (TPA) is used to obtain a series of parameters, which include hardness, adhesiveness and so on, to evaluate the texture properties of muscle by simulating human oral movements [37]. Texture characteristics are closely related to the acceptability of consumers [38]. Crispy grass carp (*Ctenopharyngodon idellus* C. ET V), belonging to freshwater fish, is popular with consumers because of its high crispness, hardness and chewiness of muscle [38]. In the present research, compared with the control group, the 4% HC and 6% HC groups have significantly higher muscle chewiness, and the 6% HC group has significantly higher muscle hardness. The increased hardness in 6% HC group might be linked to the increased myofiber density. The higher the myofiber density, the harder the muscle [22]. In the present experiment, the myofiber density in 6% HC group was highest and significantly higher than that in other HC groups. In addition to this reason, the increase in muscle hardness and chewiness might be related to the increase in muscle collagen cross-linking. The content of hydroxylysine in hydrolyzed collagen (1.38%) was much higher than that in fish meal (0.39%). Hydroxylysine has the function of forming collagen cross-linking, which improves the mechanical, thermic stableness and tractile intensity of collagen fiber [39]. The hardness of Atlantic salmon fillets was positively correlated with cross-linking concentration [40]. Compared with the control group, all HC groups have significantly lower muscle adhesiveness. Adhesiveness is inversely proportional to cell binding force [41]. The decrease in muscle adhesiveness might be related to the increase in intercellular binding force by hydrolyzed collagen. Muscle pH is related to glycolysis, fatty acid composition and some biological reactions, and the decrease in pH will lead to a soft texture [21]. In the present research, no significant difference was detected in muscle pH, and the muscle chewiness and myofiber density increased significantly when the proportion of fish meal replaced with hydrolyzed collagen reached 4%, suggesting that hydrolyzed collagen had the function of improving muscle texture of the triploid fish, which will make it more acceptable to customers.

Superoxide dismutase (SOD), a kind of metalloenzyme, is capable of catalyzing superoxide radicals (O_2_^•−^) into hydrogen peroxide (H_2_O_2_) and oxygen (O_2_), which effectively protects the body from the damage of reactive oxygen species (ROS) [42]. Glutathione peroxidase (GSH-Px) can eliminate organic hydroperoxides [43]. SOD and GSH-Px are crucial in protecting the body from antioxidant damage. Malondialdehyde (MDA) is produced by chemical reactions and enzymatic catalysis of PUFAs, and it is a biomarker of oxidative stress [44]. Compared with the control group, the activities of serum GSH-Px in all HC groups and the serum SOD in the 6% HC group increased significantly, and the content of serum MDA in all HC groups decreased significantly. The increased serum GSH-Px and SOD activities, and the decreased MDA content might be attributed to the antioxidant function of hydrolyzed collagen [10]. Peptides isolated from gelatin hydrolysate of tilapia skin have been proven to be capable of reducing the level of intracellular ROS and increasing the expression of antioxidant factors [45]. In the present study, serum GSH-Px activity increased significantly and serum MDA content decreased significantly with hydrolyzed collagen replacement, which coincided with the former research that the GSH-Px and SOD activities in the skin of mice were significantly increased after oral administration of the diet containing collagen hydrolysate [46]. Excessive ROS would cause the oxidation of subcellular membrane and structural proteins, and ultimately affect muscle texture, water-holding capacity and other quality characteristics, which was harmful to muscle quality [47]. When common carp (*Cyprinus carpio*) was exposed to oxidative stress, the muscle physicochemical properties decreased significantly [48]. The relationship between oxidative stress and muscle quality indicated that hydrolyzed collagen improved muscle quality probably by improving the antioxidant capacity of triploid crucian carp.

Glucose is an important source of energy for physiological activities [49]. Glucose in the blood is absorbed by cells in the liver and muscle under the action of insulin, and stored in the form of glycogen [50]. Hepatic glycogen is crucial for maintaining normal glucose homeostasis in the body, and it is decomposed to keep the blood glucose level in starvation in a normal range, and the content of hepatic glycogen is related to metabolic pathways such as gluconeogenesis, glycogenolysis, glycogen synthesis, glycolysis [51]. The serum glucose content decreased significantly when the proportion of fish meal replaced with hydrolyzed collagen reached 4%. The decreased serum glucose content might be attributed to the increased expression level of *IGF-1* in the liver. In the present experiment, the expression level of *IGF-1* in the liver increased significantly when the proportion of hydrolyzed collagen replacing fish meal reached 2%. Insulin-like growth factor-1, a peptide hormone, has similar amino acid sequences and functions to insulin, and it can promote glucose uptake in peripheral tissues [52]. In addition, the decreased serum glucose content in 4% HC and 6% HC groups might also be related to the enhancement of antioxidant capacity. In the present research, the serum GSH-Px activity in all HC groups increased significantly, and the serum MDA content decreased significantly compared with the control group. There is a tight relationship between oxidative stress and insulin resistance, a situation in which cells failed to utilize insulin normally, and increased GSH-Px activity helps the body to resist oxidative stress [50,53]. Glycogen phosphorylase (*GPase*) is vital in the process of glycogen decomposition, which releases glucose-1-phosphate (G-1-P) by cleaving α-1,4 glycosidic bridges, and G-1-P participates in the glycolytic pathway by conversion to glucose-6-phosphate (G-6-P) [54]. Glucokinase (*GK*) is the first enzyme in liver glycolysis, and it is also the speed-limiting enzyme, which catalyzes the conversion of glucose into G-6-P [55]. The decreased expression levels of *GPase* and *GK* in the liver might be attributed to the decreased hepatic glycogen content, resulting in the decreased activities of glycogen decomposition and glycolysis-related enzymes, but the mechanism of hepatic glycogen decrease needs to be further studied. High glucose level may stimulate vascular cells to produce ROS through the activation of NAD(P)H oxidase, which depends on protein kinase C (PKC) pathway, and ultimately affect the quality of fish [47,56]. In the present experiment, the decrease in serum glucose content may be beneficial in improving the muscle quality of fish to some extent.

Lipids are a class of organic molecules, including triglycerides, sterols, and so on, and they are crucial in energy storage and cell signal transmission [49]. When the lipid level in the body is too high, there may be various diseases, such as hypertriglyceridemia [57]. Lipids cannot flow freely in the blood because of their insolubility in water, and lipoproteins (such as HDL-C, LDL-C, very-low-density lipoproteins (VLDLs), and chylomicrons) are the main carriers of lipids transported in the blood [49]. Cholesterol, a kind of sterol, is mainly synthesized in the liver and is the precursor of steroid hormones, bile acids, etc. [58]. Low-density lipoprotein (LDL) is the main carrier of cholesterol transportation from the liver to body tissues, and a high level of LDL in serum will greatly increase the risk of arterial disease [58]. High-density lipoprotein (HDL) is the main carrier of cholesterol transportation from organism tissues to the liver, and HDL is decomposed in the liver or excreted as waste, which is essential to prevent the body from suffering from arterial diseases [58,59]. In the present experiment, the content of serum triglyceride and total cholesterol decreased significantly with hydrolyzed collagen replacement. Serum LDL-C content and liver TG content decreased significantly when the proportion of hydrolyzed collagen replacing fish meal reached 4% and 6%, respectively. The decrease in lipid content might be attributed to the increased expression levels of genes related to lipolysis and the decreased expression levels of genes related to lipogenesis in the liver. The *HADH*, *LPL* and *CPT 1* genes are related to lipolysis. Lipoprotein lipase is in the position to hydrolyze triglycerides in chylomicrons and VLDL [60]. Both Hydroxyacyl-coenzyme A dehydrogenase and carnitine O-palmitoyltransferase 1 catalyze the β-oxidation of fatty acids [61,62]. The *FAS* and *SREBP 1* genes are related to fat synthesis. Long-chain fatty acids, the principal component of triglyceride, are synthesized under the catalysis of fatty acid synthase [60]. The synthesis of cholesterol and fatty acids is controlled by the transcription factor of SREBP [63], of which SREBP-1 promotes the expression of adipogenic genes [60]. In the present research, the expression levels of *HADH*, *LPL* and *CPT 1* in the liver increased significantly, and the expression levels of *FAS* and *SREBP 1* decreased significantly with hydrolyzed collagen replacement, indicating that hydrolyzed collagen decreased the body lipid content probably by promoting fat decomposition and inhibiting fat synthesis. In addition, the decrease in serum lipid content might be related to the decrease in SFAs content and the increase in PUFAs content in muscle. Previous studies have proved that the higher the SFAs content, the higher the LDL-C content in the body [36], and the n-3 PUFAs and n-6 PUFAs are effective in reducing LDL-C and T-CHO content [33]. In the present experiment, the content of serum TG, T-CHO and LDL-C decreased significantly with fish meal replaced with hydrolyzed collagen, and the results coincided with the former research [8], which indicated that the levels of plasma total lipids and triglycerides decreased significantly in mice treated with oral collagen hydrolysates.

## 5. Conclusions

It can be concluded that when hydrolyzed collagen replaced 2% fish meal, it did not significantly impact the growth and intestinal digestive enzymes activities of triploid crucian carp. When the ratio of fish meal replaced with hydrolyzed collagen reached 4%, SGR decreased, and muscle quality and glycolipid metabolism were improved. The maximum proportion of hydrolyzed collagen replacing fish meal should not exceed 4%. Hydrolyzed collagen improved muscle quality by increasing muscle chewiness, myofiber density and the content of total umami amino acids, ALA, LA and n-6 PUFAs in muscle, as well as increasing serum antioxidant capacity. Hydrolyzed collagen decreased serum glucose probably by up-regulating the expression level of *IGF-1*, and decreased body lipid content probably by up-regulating the expression levels of lipolytic enzyme genes (*HAHD*, *LPL*, *CPT 1*) and down-regulating the expression levels of adipogenic enzyme genes (*FAS*, *SREBP 1*). These studies indicated that hydrolyzed collagen had the effect of improving muscle quality and glycolipid metabolism of fish, which proved the feasibility of fish meal replaced with hydrolyzed collagen in fish feed.

## Figures and Tables

**Table 1:** Formulation and proximate analysis of experimental diets (% dry matter).

	Control	2% HC	4% HC	6% HC
Fish meal	9.00	6.00	3.00	0.00
Soybean meal	26.00	26.00	26.00	26.00
Rapeseed meal	23.00	23.00	23.00	23.00
Wheat flour	27.65	27.65	28.65	29.65
DDGS ^a^	7.00	7.00	7.00	7.00
Soybean oil	4.00	5.00	5.00	5.00
Premix ^b^	1.00	1.00	1.00	1.00
Choline chloride	0.20	0.20	0.20	0.20
Ethoxyquin	0.10	0.10	0.10	0.10
Antioxidants	0.05	0.05	0.05	0.05
Calcium dihydrogen	2.00	2.00	2.00	2.00
Hydrolyzed collagen ^c^	0.00	2.00	4.00	6.00
Proximate analysis				
Crude protein	31.77	32.05	32.84	31.12
Crude lipid	8.41	8.25	8.97	8.17

^a.^ DDGS: Distillers dried grains with solubles. ^b.^ The premix was provided by MGOTer Biotechnology Co., Ltd. (Qingdao, China). Each kilogram of the premix contains FeSO_4_·H_2_O, 400 mg; KCl, 200 mg; ZnSO_4_·H_2_0, 400 mg; KI (1%), 60 mg; MgSO_4_·H_2_O, 2000 mg; CoCl_2_·6H_2_O (1%), 50 mg; CuSO_4_·5H_2_O, 30 mg; MnSO_4_·H_2_O, 150 mg; Na_2_SeO_3_·5H_2_0 (1%), 65 mg; Zeolite powder, 3645.85 mg; Vitamin B_1_, 12 mg; Vitamin B_2_, 12 mg; Vitamin B_6_, 8 mg; Vitamin B_12_, 0.05 mg; Vitamin K_3_, 8 mg; Inositol, 100 mg; Folic acid, 5 mg; Vitamin E, 50 mg; Biotin, 0.8 mg; Niacin, 50 mg; Pantothenic acid, 40 mg; Vitamin A, 25 mg; VCP 1, 5 mg; Vitamin C, 100 mg; Ethoxyquin, 150 mg; and Wheat flour, 2434.15 mg. ^c.^ Hydrolyzed collagen (purity: 99%) was from Baichuan Biotechnology Co., Ltd. (Xi’an, China).

**Table 2:** Comparison of proximate composition between hydrolyzed collagen and fish meal (% wet basis).

	Moisture (%)	Crude Protein (%)	Crude Lipid (%)
Hydrolyzed collagen	5.19	88.51	6.91
Fish meal	7.70	55.25	10.60

**Table 3:** Comparison of amino acids between hydrolyzed collagen and fish meal (% dry matter).

	Hydrolyzed Collagen	Fish Meal
Methionine	0.62	1.02
Valine	2.49	3.00
Lysine	3.84	4.89
Isoleucine	1.26	2.51
Phenylalanine	1.97	2.73
Leucine	2.69	4.41
Threonine	2.05	2.69
Histidine	0.62	1.26
Arginine	7.64	3.70
Glutamate	9.80	8.47
Glycine	22.20	4.13
Alanine	9.88	4.08
Cysteine	0.00	0.24
Tyrosine	0.34	1.74
Aspartic acid	5.41	5.75
Serine	3.01	2.40
Proline	11.74	2.35
Hydroxyproline	6.32	0.34
Hydroxylysine	1.38	0.39
EAA ^a^	23.18	26.21
NEAA ^b^	70.08	29.89
TAA ^c^	93.26	56.10
EAA/TAA	24.86	46.72
NEAA/TAA	75.14	53.28

^a.^ EAA: essential amino acid. ^b.^ NEAA: non-essential amino acid. ^c.^ TAA: total amino acid.

**Table 4:** Amino acid composition of diets (% dry matter).

	Control	2% HC	4% HC	6% HC
Methionine	0.43	0.42	0.37	0.34
Valine	1.56	1.61	1.47	1.41
Lysine	1.88	1.89	1.83	1.63
Isoleucine	1.32	1.33	1.20	1.12
Phenylalanine	1.52	1.59	1.50	1.39
Leucine	2.51	2.55	2.32	2.19
Threonine	1.32	1.35	1.24	1.18
Histidine	0.83	0.83	0.78	0.71
Arginine	1.89	2.05	1.99	2.04
Glutamate	6.33	6.65	6.23	6.00
Glycine	1.62	2.09	2.36	2.69
Alanine	1.66	1.83	1.82	1.87
Cysteine	0.41	0.44	0.39	0.41
Tyrosine	0.91	0.95	0.81	0.81
Aspartic acid	2.75	2.84	2.63	2.43
Serine	1.53	1.61	1.51	1.48
Proline	2.06	2.37	2.45	2.64
Hydroxyproline	0.19	0.41	0.60	0.80
EAA ^a^	13.25	13.61	12.70	11.99
NEAA ^b^	17.46	19.18	18.80	19.15
TAA ^c^	30.71	32.78	31.50	31.14

^a.^ EAA: essential amino acid. ^b.^ NEAA: non-essential amino acid. ^c.^ TAA: total amino acid.

**Table 5:** Real-time PCR primer sequences.

Gene	Genbank Accession Number		Primer Sequence (5′-3′)	Annealing Temperature (°C)	Product Length
*β-actin*	AB039726.2	F	TCTACAACGAGCTGCGTGTTG	55.0	80
		R	CCTGTTGGCTTTGGGATTGA		
*GK*	XM_026229812.1	F	CGAAGAGCCAACAGGTATA	51.0	143
		R	AAAGGGAAGTTGTCCATCA		
*PK*	XM_026227921.1	F	TCGGCTCATTTATTGTCTCG	55.0	134
		R	ATGTCAGTGGGCGGAGTT		
*G6Pase*	XM_026223099.1	F	CTTCATCGCCGCTCATTT	51.0	102
		R	CTGGCACTGTAGATCCATTTCT		
*HIF1α*	XM_026233707.1	F	CTCTGCTTCTGATCGTGGTC	52.7	176
		R	GGGTCTTTGCCTCGGTAT		
*GSase*	XM_026236547.1	F	TGCGGATGAGTCGTGTAA	53.0	234
		R	GGGTAACGGAAGCCTTGT		
*GPase*	XM_026278074.1	F	CCGACCCTAAGCGTGTAT	52.9	188
		R	TAATCTTCCCAGCCCTCC		
*IGF-1*	AF001005.1	F	TGCGTCCTCGCGTTGACT	57.2	169
		R	CCACGATGCCACGGTTGT		
*ACOX1*	XM_026213979.1	F	TGACTACCGTGATGAGATG	50.2	105
		R	TCAGTATGGTTCAGAGGG		
*HADH*	XM_026203611.1	F	TGCGTCTGTAGTTCAGGG	52.1	186
		R	CCACCAAAGCGATCTAAT		
*LPL*	FJ204474.1	F	GCAGCATTGGGATTCAGA	51.9	152
		R	CTCATGGGAGCACTTCAC		
*CPT 1*	XM_026200694.1	F	CGAGCAACAGATTCAGCG	53.4	187
		R	ATCCAGGGCAACAAAGAA		
*FAS*	XM_026234785.1	F	CGTGGGCATCAACTCATT	53.5	191
		R	GGTAGCTTGGGTTATCCTTAT		
*ACC*	XM_026210883.1	F	GTTACGCCGCTGGTTTGT	52.6	206
		R	CGGTCACTTCTGGGTTGG		
*SREBP 1*	XM_026207478.1	F	TGGGTCATCGTTTCTTTGTG	55.5	186
		R	CATCAGTGAGGGTCTTGTCG		

**Table 6:** Effects of hydrolyzed collagen replacing fish meal on growth and morphometric indexes of triploid crucian carp.

	Control	2% HC	4% HC	6% HC	*p* Value		
					ANOVA	Linear Trend	Quadratic Trend
FBW (g)	47.83 ± 1.85 ^b^	41.16 ± 2.33 ^b^	31.85 ± 0.91 ^a^	31.64 ± 2.44 ^a^	0.001	0.000	0.001
WGR	380.74 ± 18.59 ^b^	309.28 ± 23.17 ^b^	217.30 ± 9.08 ^a^	216.84 ± 24.41 ^a^	0.001	0.000	0.001
SGR (%/d)	2.38 ± 0.06 ^b^	2.13 ± 0.08 ^b^	1.75 ± 0.04 ^a^	1.74 ± 0.11 ^a^	0.001	0.000	0.001
HSI (%)	2.51 ± 0.16	2.51 ± 0.21	2.16 ± 0.24	2.41 ± 0.27	0.654	0.510	0.807
VSI (%)	15.59 ± 0.38	15.22 ± 0.31	14.36 ± 0.63	14.02 ± 0.64	0.165	0.033	0.082
CF (g/cm^3^)	3.07 ± 0.04	3.05 ± 0.06	2.90 ± 0.08	2.98 ± 0.03	0.164	0.119	0.311

Values are presented as mean ± SE (*n* = 3). Different superscript letters of peers indicate significant differences (*p* < 0.05).

**Table 7:** Effects of hydrolyzed collagen replacing fish meal on muscle nutrition components of triploid crucian carp (% wet basis).

	Control	2% HC	4% HC	6% HC	*p* Value		
					ANOVA	Linear Trend	Quadratic Trend
Moisture (%)	75.92 ± 0.30	75.12 ± 0.48	75.57 ± 0.30	75.37 ± 0.28	0.468	0.283	0.401
Crude protein (%)	17.36 ± 0.36	17.70 ± 0.40	17.49 ± 0.29	17.17 ± 0.22	0.706	0.843	0.476
Crude lipid (%)	2.32 ± 0.08	2.02 ± 0.18	2.32 ± 0.12	2.55 ± 0.09	0.096	0.447	0.043

Values are presented as mean ± SE (*n* = 3).

**Table 8:** Effects of hydrolyzed collagen replacing fish meal on muscle free amino acid of triploid crucian carp (mg/100 g, wet basis).

	Control	2% HC	4% HC	6% HC
Glutamate	6.91 ± 0.20 ^a^	7.68 ± 0.31 ^ab^	8.74 ± 0.18 ^c^	8.30 ± 0.20 ^bc^
Aspartic acid	2.24 ± 0.09	2.26 ± 0.02	2.04 ± 0.09	2.06 ± 0.01
Glycine	42.10 ± 0.19 ^a^	43.23 ± 0.25 ^a^	40.49 ± 1.41 ^a^	57.70 ± 0.60 ^b^
Alanine	18.77 ± 0.07 ^b^	21.61 ± 0.12 ^c^	15.34 ± 0.58 ^a^	15.86 ± 0.15 ^a^
Lysine	20.34 ± 0.09 ^a^	26.09 ± 0.17 ^c^	22.54 ± 0.77 ^b^	26.19 ± 0.25 ^c^
Serine	3.86 ± 0.11 ^a^	5.70 ± 0.04 ^b^	5.77 ± 0.19 ^b^	8.55 ± 0.08 ^c^
Proline	28.20 ± 0.13 ^d^	14.64 ± 0.09 ^c^	8.34 ± 0.14 ^b^	6.41 ± 0.09 ^a^
Threonine	10.44 ± 0.04	11.00 ± 0.07	10.43 ± 0.35	11.05 ± 0.11
Arginine	25.52 ± 0.08 ^d^	16.57 ± 0.11 ^c^	15.03 ± 0.62 ^b^	10.99 ± 0.10 ^a^
Leucine	4.30 ± 0.13 ^bc^	3.68 ± 0.02 ^a^	3.92 ± 0.17 ^ab^	4.46 ± 0.07 ^c^
Methionine	1.10 ± 0.04 ^a^	1.16 ± 0.01 ^a^	1.20 ± 0.05 ^a^	1.35 ± 0.01 ^b^
Valine	2.92 ± 0.08 ^ab^	2.67 ± 0.02 ^a^	2.74 ± 0.12 ^ab^	3.06 ± 0.02 ^b^
Tyrosine	2.08 ± 0.08 ^b^	2.37 ± 0.01 ^c^	1.99 ± 0.09 ^b^	1.46 ± 0.01 ^a^
Isoleucine	2.31 ± 0.07 ^b^	1.92 ± 0.01 ^a^	2.14 ± 0.09 ^ab^	2.27 ± 0.03 ^b^
Phenylalanine	1.42 ± 0.04 ^b^	1.41 ± 0.01 ^b^	1.23 ± 0.05 ^a^	1.24 ± 0.01 ^a^
Histidine	327.54 ± 1.28 ^a^	373.81 ± 2.26 ^c^	372.26 ± 1.58 ^c^	351.95 ± 3.33 ^b^
Umami AA ^(a)^	9.15 ± 0.26 ^a^	9.94 ± 0.29 ^ab^	10.78 ± 0.26 ^b^	10.36 ± 0.21 ^b^
Sweet AA ^(b)^	123.71 ± 0.55 ^b^	122.28 ± 0.73 ^b^	102.92 ± 3.14 ^a^	125.77 ± 1.28 ^b^
Bitter AA ^(c)^	33.85 ± 0.27 ^c^	24.07 ± 0.16 ^b^	22.89 ± 0.95 ^b^	19.87 ± 0.19 ^a^
TAA ^(d)^	500.06 ± 1.78 ^a^	535.81 ± 3.45 ^b^	514.21 ± 4.43 ^a^	512.92 ± 5.05 ^a^

Values are presented as mean ± SE (*n* = 3). Different superscript letters of peers indicate significant differences (*p* < 0.05). ^(a)^ Umami AA: umami amino acid (Asp, Glu). ^(b)^ Sweet AA: sweet amino acid (Gly, Ala, Lys, Ser, Pro, Thr). ^(c)^ Bitter AA: bitter amino acid (Arg, Leu, Met, Val). ^(d)^ TAA: total amino acid.

**Table 9:** Effects of hydrolyzed collagen replacing fish meal on muscle fatty acid composition of triploid crucian carp (g/100 g total fatty acids).

	Control	2% HC	4% HC	6% HC
C14:0	0.68 ± 0.01 ^b^	0.61 ± 0.01 ^a^	0.60 ± 0.02 ^a^	0.55 ± 0.02 ^a^
C16:0	17.22 ± 0.16	16.59 ± 0.19	16.66 ± 0.27	16.53 ± 0.14
C18:0	7.88 ± 0.13 ^c^	7.25 ± 0.12 ^ab^	7.06 ± 0.09 ^a^	7.72 ± 0.10 ^bc^
C16:1 n-7	2.78 ± 0.08 ^b^	2.61 ± 0.02 ^ab^	2.55 ± 0.05 ^a^	2.43 ± 0.03 ^a^
C18:1 n-9	43.37 ± 0.40	42.89 ± 0.55	41.64 ± 1.02	44.11 ± 0.4
C20:1 n-9	2.62 ± 0.03	2.48 ± 0.06	2.45 ± 0.12	2.49 ± 0.08
C18:3 n-3	1.81 ± 0.02 ^a^	2.06 ± 0.06 ^b^	2.08 ± 0.04 ^b^	1.98 ± 0.05 ^ab^
C22:5 n-3	0.24 ± 0.02 ^b^	0.23 ± 0.01 ^b^	0.27 ± 0.03 ^b^	0.00 ± 0.00 ^a^
C22:6 n-3	2.53 ± 0.31 ^b^	1.79 ± 0.29 ^ab^	1.80 ± 0.29 ^ab^	0.81 ± 0.11 ^a^
C18:2 n-6	18.45 ± 0.14 ^a^	20.89 ± 0.34 ^b^	21.66 ± 0.37 ^b^	20.67 ± 0.29 ^b^
C18:3 n-6	0.26 ± 0.01 ^a^	0.33 ± 0.01 ^ab^	0.39 ± 0.05 ^b^	0.35 ± 0.01 ^ab^
C20:3 n-6	1.15 ± 0.09	1.18 ± 0.07	1.37 ± 0.08	1.21 ± 0.08
C20:4 n-6	1.01 ± 0.12	1.10 ± 0.08	1.47 ± 0.15	1.13 ± 0.12
SFAs ^(a)^	25.78 ± 0.07 ^b^	24.45 ± 0.25 ^a^	24.32 ± 0.19 ^a^	24.80 ± 0.07 ^a^
MUFAs ^(b)^	48.77 ± 0.44	47.97 ± 0.59	46.64 ± 1.16	49.03 ± 0.46
PUFAs ^(c)^	25.44 ± 0.40 ^a^	27.58 ± 0.48 ^ab^	29.04 ± 0.99 ^b^	26.17 ± 0.42 ^a^
n-3 PUFAs ^(d)^	4.57 ± 0.30 ^b^	4.09 ± 0.28 ^b^	4.16 ± 0.35 ^b^	2.80 ± 0.11 ^a^
n-6 PUFAs ^(e)^	20.87 ± 0.11 ^a^	23.49 ± 0.32 ^b^	24.89 ± 0.64 ^b^	23.37 ± 0.31 ^b^
n-9 MUFAs ^(f)^	45.99 ± 0.42	45.37 ± 0.61	44.09 ± 1.13	46.60 ± 0.45

Values are presented as mean ± SE (*n* = 3). Different superscript letters of peers indicate significant differences (*p* < 0.05). ^(a)^ SFAs: Saturated fatty acids (C14:0, C16:0, C18:0). ^(b)^ MUFAs: Monounsaturated fatty acids (C16:1 n-7, C18:1 n-9, C20:1 n-9). ^(c)^ PUFAs: Polyunsaturated fatty acids (C18:3 n-3, C22:5 n-3, C22:6 n-3, C18:2 n-6, C18:3 n-6, C20:3 n-6, C20:4 n-6). ^(d)^ n-3 PUFAs: n-3 Polyunsaturated fatty acids (C18:3 n-3, C22:5 n-3, C22:6 n-3). ^(e)^ n-6 PUFAs: n-6 Polyunsaturated fatty acids (C18:2 n-6, C18:3 n-6, C20:3 n-6, C20:4 n-6). ^(f)^ n-9 MUFAs: n-9 Monounsaturated fatty acids (C18:1 n-9, C20:1 n-9).

**Table 10:** Effects of hydrolyzed collagen replacing fish meal on muscle texture indexes, pH value, myofiber density and diameter of triploid crucian carp.

	Control	2% HC	4% HC	6% HC	*p* Value		
					ANOVA	Linear Trend	Quadratic Trend
Hardness (N)	5.29 ± 0.36 ^a^	6.10 ± 0.28 ^a^	5.22 ± 0.70 ^a^	9.73 ± 0.65 ^b^	0.001	0.044	0.011
Adhesiveness (N.mm)	0.07 ± 0.01 ^c^	0.04 ± 0.00 ^b^	0.02 ± 0.00 ^a^	0.01 ± 0.00 ^a^	0.000	0.000	0.000
Cohesiveness	0.38 ± 0.03	0.36 ± 0.03	0.41 ± 0.03	0.43 ± 0.03	0.402	0.257	0.254
Springiness (mm)	1.10 ± 0.05	1.10 ± 0.03	1.25 ± 0.07	1.19 ± 0.03	0.160	0.121	0.304
Chewiness (mJ)	2.05 ± 0.04 ^a^	2.03 ± 0.12 ^a^	4.14 ± 0.22 ^b^	5.18 ± 0.18 ^c^	0.000	0.001	0.000
pH	6.68 ± 0.07	6.59 ± 0.04	6.63 ± 0.07	6.61 ± 0.05	0.758	0.387	0.602
Myofiber density (cell/mm^2^)	112.81 ± 5.22 ^a^	127.71 ± 3.73 ^a^	166.97 ± 7.05 ^b^	201.71 ± 8.02 ^c^	0.000	0.000	0.000
Myofiber diameter (µm)	87.74 ± 2.52 ^b^	81.19 ± 2.95 ^b^	71.95 ± 1.20 ^a^	64.22 ± 2.29 ^a^	0.000	0.000	0.000

Values are presented as mean ± SE (*n* = 3, except myofiber density and myofiber diameter (*n* = 6)). Different superscript letters of peers indicate significant differences (*p* < 0.05).

**Table 11:** Effects of hydrolyzed collagen replacing fish meal on serum, intestinal and liver enzymes activities of triploid crucian carp.

	Control	2% HC	4% HC	6% HC	*p* Value		
					ANOVA	Linear Trend	Quadratic Trend
Serum antioxidation							
CAT (U/mL)	4.18 ± 1.00	4.38 ± 0.52	4.18 ± 0.70	3.43 ± 0.04	0.760	0.542	0.554
SOD (U/mL)	76.14 ± 2.47 ^a^	70.78 ± 0.80 ^a^	74.19 ± 1.15 ^a^	83.93 ± 0.31 ^b^	0.000	0.203	0.000
GSH-Px (U/mL)	210.11 ± 11.54 ^a^	273.1 ± 6.61 ^b^	273.56 ± 5.17 ^b^	252.87 ± 10.34 ^b^	0.001	0.005	0.000
MDA (nmol/mL)	6.94 ± 0.36 ^c^	5.25 ± 0.19 ^b^	5.76 ± 0.18 ^b^	3.60 ± 0.16 ^a^	0.000	0.000	0.000
Intestinal digestive enzymes							
α-Amylase (U/mgprot)	27.29 ± 2.66 ^b^	24.31 ± 0.92 ^ab^	19.32 ± 1.69 ^a^	17.79 ± 0.94 ^a^	0.015	0.002	0.006
Trypsin (U/mgprot)	726.99 ± 33.92 ^b^	844.34 ± 31.30 ^b^	556.33 ± 13.48 ^a^	551.11 ± 41.31 ^a^	0.000	0.075	0.017
Lipase (U/gprot)	1.09 ± 0.04 ^b^	0.93 ± 0.02 ^b^	0.75 ± 0.05 ^a^	0.67 ± 0.03 ^a^	0.000	0.000	0.000
Glucose and glycogen content							
Serum glucose (mmol/L)	9.23 ± 0.06 ^b^	9.88 ± 0.29 ^b^	8.11 ± 0.06 ^a^	7.42 ± 0.18 ^a^	0.000	0.024	0.000
Liver glycogen (mg/g)	63.53 ± 5.40 ^b^	51.04 ± 3.91 ^ab^	40.58 ± 4.48 ^a^	39.64 ± 2.98 ^a^	0.006	0.000	0.003
Lipid content							
Serum TG (mmol/L)	2.43 ± 0.18 ^b^	1.41 ± 0.09 ^a^	1.40 ± 0.10 ^a^	1.41 ± 0.08 ^a^	0.000	0.000	0.000
Serum T-CHO (mmol/L)	7.09 ± 0.14 ^b^	6.32 ± 0.07 ^a^	5.75 ± 0.21 ^a^	6.01 ± 0.23 ^a^	0.001	0.000	0.001
Serum HDL-C (mmol/L)	4.04 ± 0.18	3.89 ± 0.15	3.95 ± 0.16	3.76 ± 0.21	0.724	0.318	0.596
Serum LDL-C (mmol/L)	4.55 ± 0.07 ^b^	4.10 ± 0.21 ^ab^	4.23 ± 0.10 ^ab^	3.91 ± 0.06 ^a^	0.021	0.004	0.019
Liver TG (mmol/gprot)	0.88 ± 0.06 ^b^	0.56 ± 0.08 ^ab^	0.35 ± 0.02 ^a^	0.50 ± 0.13 ^a^	0.010	0.004	0.009
Liver T-CHO (mmol/gprot)	0.01 ± 0.00	0.01 ± 0.00	0.01 ± 0.00	0.01 ± 0.00	0.131	0.735	0.650

Values are presented as mean ± SE (*n* = 3). Different superscript letters of peers indicate significant differences (*p* < 0.05).

## Data Availability

The data are contained within the article.
